# Tortuous Common Carotid Artery: A Report of Four Cases Observed in Cadaveric Dissections

**DOI:** 10.1155/2016/2028402

**Published:** 2016-10-13

**Authors:** Joe Iwanaga, Koichi Watanabe, Saga Tsuyoshi, Yoko Tabira, Koh-ichi Yamaki

**Affiliations:** Department of Anatomy, Kurume University School of Medicine, Kurume, Fukuoka 830-0011, Japan

## Abstract

A tortuous common carotid artery poses a high risk of injury during tracheotomy. Preoperative diagnosis is therefore important to avoid serious complications. We found four cases of tortuous common carotid artery during an anatomical dissection course for students. The first case was a 91-year-old woman who had bilateral tortuous common carotid arteries without arteriosclerosis. Case 2 was a 78-year-old woman who had bilateral tortuous common carotid arteries without arteriosclerosis. Case 3 was an 86-year-old woman who died from bladder cancer and who also had a right tortuous common carotid artery without arteriosclerosis. Case 4 was an 89-year-old woman who had bilateral tortuous common carotid arteries and a tortuous brachiocephalic artery with severe arteriosclerosis. Case 4 was also examined using computed tomography to evaluate the arteriosclerosis. Computed tomography revealed severe calcification of the vascular wall, which was confirmed in the aortic arch and origins of its branches. In all four cases, the tortuosity was located below the level of the thyroid gland. Based on prior study results indicating that fusion between the carotid sheath and visceral fascia was often evident at the level of the thyroid gland, we speculated that the major region in which tortuosity occurs is at the same level or inferior to the level of the thyroid gland.

## 1. Introduction

Tortuous common carotid artery (CCA) is associated with a risk of injury during surgical procedures in the anterior cervical region, such as tracheotomy [1, 2]. Therefore, the preoperative examination is important. Computed tomographic angiography (CTA) can provide an accurate diagnosis [3]. For patients with a tortuous CCA who require tracheotomy, evaluation of the three-dimensional relation between the CCA and the thyroid gland is of great interest. There have been few reports, however, that included an accurate description of those structures.

We describe four cases of tortuous CCA that were found among 32 cadavers during a gross anatomical dissection course for students in 2015 to explore the cause of tortuous CCA and its relation with the thyroid gland. To clarify the appearance of the tortuous CCA more definitively, one of the four cases (with bilateral severe tortuous CCA and arteriosclerosis) underwent radiological examination as well. This study was performed in keeping with the requirements of the Declaration of Helsinki.

## 2. Case Reports

**Figure 1:**
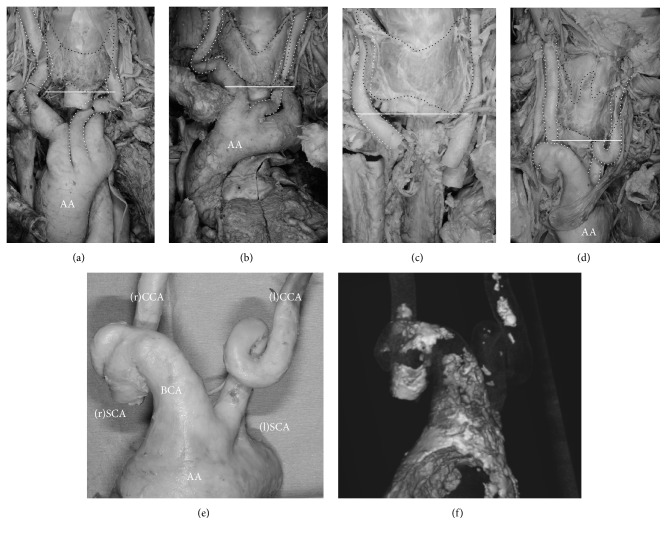
Four cases of tortuous common carotid artery (CCA) reported in the present study. Black dotted lines indicate the thyroid glands. White dotted lines indicate the common carotid arteries. White lines show the inferior border of the thyroid glands. (a) Case 1. Bilateral tortuous CCAs ascend posterior to the thyroid gland. The left side is especially tortuous. (b) Case 2. Bilateral tortuous CCAs ascend posterior to the thyroid gland. The right side is especially tortuous. (c) Case 3. Tortuous CCA on the right side ascends posterior to the thyroid gland. The aortic arch had been removed during dissection. (d) Case 4. Bilateral tortuous CCAs ascend posterior to the thyroid gland with a 360° turn. The brachiocephalic trunk is also tortuous and hypertrophied. (e) Case 4. Aortic arch and its branches after removal from the body. The vascular wall is highly calcified and has lost its elasticity. (f) Case 4. Computed tomography (CT) image shows the calcified part of the aortic arch and its branches. White parts, which had high CT values, are suspected to be calcified. AA: aortic arch; BCA: brachiocephalic artery; CCA: common carotid artery; SCA: subclavian artery.

Case 1 was a 91-year-old woman who died from lung cancer and who also had bilateral tortuous CCAs (Figure 1(a)). The position of the right tortuosity was at the mid-thyroid level and posterior to the gland. The position of the left tortuosity was slightly lower than and lateral to the inferior border of the thyroid gland. The left CCA was more tortuous than that on the right. There was no evidence of arteriosclerosis.

Case 2 was a 78-year-old woman who died from multiple organ failure. She was also found to have bilateral tortuous CCAs (Figure 1(b)). The position of the right tortuosity was at the mid-thyroid level and lateral to the gland. The position of the left tortuosity was lower than the inferior border of the thyroid gland. The right CCA was more tortuous than that on the left. There was no evidence of arteriosclerosis.

Case 3 was an 86-year-old woman who died from bladder cancer and who had a right tortuous CCA (Figure 1(c)). The position of the tortuosity was at the level of the inferior border of, and posterior to, the thyroid gland. There was no evidence of arteriosclerosis.

Case 4 was an 89-year-old woman who died of old age. At postmortem evaluation, she was found to have bilateral tortuous CCAs and a tortuous brachiocephalic artery (BCA; Figure 1(d)). The right and left tortuosities were both located below the level of the inferior border of the thyroid gland. The BCA protruded and was positioned anterior to the thyroid gland. Palpation of the aortic arch and its branches led to a suspicion of severe arteriosclerosis, so CT was performed after resection. The CT images indicated arteriosclerotic changes in the aortic arch, BCA, and both CCAs (Figures 1(e) and 1(f)).

## 3. Discussion

Tortuous CCA is generally asymptomatic, although occasionally a patient becomes aware of a pulsating mass in the neck region, indicating tortuous CCA. Tortuous CCA is also sometimes found incidentally during imaging. An arterial vascular lesion due to a tortuous CCA aneurysm could affect blood flow to the brain and may result in cerebrovascular disease [4]. Rare cases of a tortuous CCA causing dysphagia [5] and a tortuous CCA presenting as a pediatric submandibular mass lesion [6] have been reported.

Tortuous CCA is sometimes found in the clinical setting, more often in women than men. It appears more frequently on the right side than on the left. Risk factors are old age, obesity, arteriosclerosis, hypertension, and heart enlargement [7]. The ages of the present four cases were 78–91 years, and all were women. Three cases showed tortuous CCA without arteriosclerosis, and one case had severe tortuous CCA and arteriosclerosis. Calcification of the vascular wall in case 4 was found especially in the aortic arch and at the origins of its branches, not in the middle of the tortuosity.

**Figure 2:**
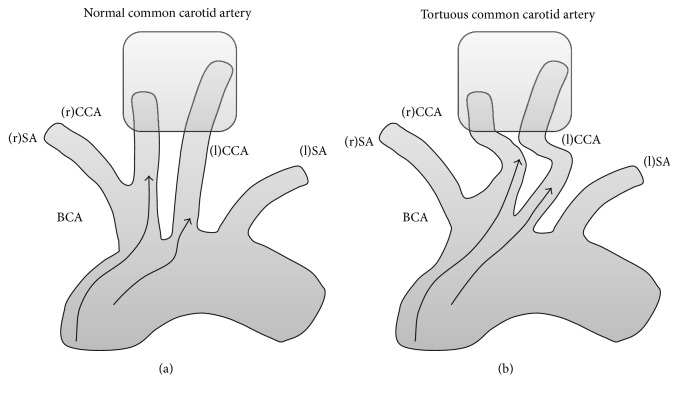
Relations among the carotid sheath, visceral fascia, and tortuosity. The square indicates the region of tight fusion between the carotid sheath and cervical visceral fascia. (a) Ejection power of the heart (black arrow) directly affects the brachiocephalic artery and right and left carotid arteries. (b) Ejection power of the heart (black arrow) results in tortuosity of the brachiocephalic artery and the right and left carotid arteries. BCA: brachiocephalic artery; (l)CCA: left common carotid artery; (l)SA: left subclavian artery; (r)SA: right subclavian artery; (r)CCA: right common carotid artery.

Our findings led us to presume that arteriosclerosis was not an immediate trigger of tortuosity of the CCA, but a complication of it. We presumed that the cardiac pumping and the strength of the connection between the cervical visceral fascia and carotid sheath comprise the potential cause of tortuous CCA. Originally, cardiac pumping is stronger in patients with hypertension and cardiac hypertrophy than in healthy people. The BCA and left CCA are the vessels most commonly affected by the force of the cardiac pumping (Figure 2). The farther the vessels are from the heart, the less the pumping influences them.

Hayashi [8] reported that fusion between the carotid sheath and visceral fascia was often evident at the level of the thyroid gland. We therefore speculated that a fixed CCA does not curve at the level of the thyroid gland. In fact, the tortuous CCAs in our four cases were found at the same level as the thyroid gland or inferior to it (Figures 1(a)–1(d)). In addition, the length and direction of the ascending aorta (which were not investigated in this study) could affect the tortuosity. Hence, surgical procedures in the anterior neck region (e.g., tracheotomy) should be modified in patients who have a tortuous CCA that protrudes or is positioned higher than the normal CCA [9, 10]. Because tracheotomy in a patient with a tortuous CCA puts the patient at high risk of injury to a major artery, a three-dimensional analysis, such as CTA [3], is required when the situation allows. The consensus is that a procedure that can evaluate the relations between the tortuous CCA and cervical organs, not only morphology, is needed.
